# Expression analysis of Argonaute genes in maize (*Zea mays* L.) in response to abiotic stress

**DOI:** 10.1186/s41065-019-0102-z

**Published:** 2019-07-24

**Authors:** Lihong Zhai, Feng Teng, Kangpeng Zheng, Juan Xiao, Wenbin Deng, Wei Sun

**Affiliations:** 1 Medical College, Hubei University of Arts and Science, Xiangyang, 441053 People’s Republic of China; 2 College of Agriculture and Biology, Zhongkai University of Agriculture and Engineering, Guangzhou, 510225 People’s Republic of China

## Abstract

**Background:**

Argonaute (AGO) protein is a kind of RNA binding protein that plays an integral role in the gene-silencing pathways guided by small RNAs. But there are few studies about the regulation of AGO genes responded to diverse abiotic stress in maize.

**Results:**

In this study, we analyzed the expression of seventeen ZmAGO genes under heat, cold, salinity, drought and ABA treatments using quantitative PCR (qPCR). All *ZmAGOs* showed differential expression modes under various abiotic stress treatments. Two *ZmAGOs* (*ZmAGO1a* and *ZmAGO5d*) and other fifteen ZmAGOs exhibited specific up-regulation in response to heat separately. Several *ZmAGO* genes are very sensitive to cold stress, but many *ZmAGO* genes are slow to respond to NaCl treatment. Nine *ZmAGO* genes (*ZmAGO1f*, *ZmAGO2b*, *ZmAGO4*, *ZmAGO5a/b/c*, *ZmAGO7*, *ZmAGO9* and *ZmAGO18a/b*) presented definite up-regulation in response to drought, which were similar to the pattern of gene regulation under abscisic acid (ABA) treatment.

**Conclusions:**

Various *ZmAGO* genes respond to different abiotic stress treatments. These results provide fundamental information and insights for the further study on the role of abiotic stress resistance genes in maize and provide basis for further study on the function of AGO genes in response to abiotic stress in maize.

**Electronic supplementary material:**

The online version of this article (10.1186/s41065-019-0102-z) contains supplementary material, which is available to authorized users.

## Background

Global climate change threatens crop yield by imposing ambient pressures such as cold, drought, salinity, heat and other abiotic and biotic stresses [1]. Numerous studies indicate that small RNAs (sRNAs) have important roles in gene expression regulation during the abiotic and biotic stresses in all plants [2–6]. Argonaute (AGO) proteins are the key effectors of the RNA-induced silencing complex (RISC). The small RNAs are categorized to bind to specific AGO family proteins which then guide RISC to silence their targets through complementary base paring. Typical functions of plant RISCs include the target RNAs endonucleolytic cleavage or translational inhibition and the target DNAs methylations [7, 8].

In plants, different species encode different numbers of AGO family members. Arabidopsis possesses 10 AGOs [9], whereas there are 17 in maize [10] and 19 in rice [11]. Phylogenetically, plants AGOs are divided into three major groups: AGO1/5/10, AGO2/3/7 and AGO4/6/8/9. In addition, the grass-specific AGO18 family has an important role in viral defense and plant reproduction [12, 13]. A series of studies of *ago1* mutants indicated that AGO1 has a role in leaf polarity [14] and lateral organ development [15] of Arabidopsis, and that AGO1 plays a role in carrying out the function of most miRNAs [16]. Furthermore, AtAGO1 also functions to effectively clean up viral RNAs [17, 18]. Recent research showed that AtAGO1 binds to chromatin to promote gene expression in response to plant hormones and stresses [19]. In addition, AGO1 also interacts with chromatin at MIR161 and MIR173 loci and leads to the disassembly of the transcriptional complex, releasing short and unpolyadenylated transcripts under salt stress conditions [20]. AtAGO10 functions in inflorescence meristem and shoot apical meristem development through completely binding to miR165/166 with AtAGO1 and jointly binding to miR172 to enhance the function of AtAGO1, respectively [21–23]. Different from AtAGO1 and AtAGO10, it was discovered that AtAGO5 expressed around reproductive cells during megasporogenesis, and *ago5* mutants exhibited deficiencies in the initial stage of megagametogenesis [24]. Recent research showed that AtAGO5 expression is induced by *Potexvirus* infection and that both AtAGO2 and AtAGO5 are needed to completely limit PVX infection in Arabidopsis [25].

AtAGO2 is found to play a key role in pathogen defense and DNA repair [26–29]. Although AtAGO3 and AtAGO2 are tandem repeat genes, AtAGO3 cannot act redundantly with AtAGO2 in pathogen defense. However, AtAGO3-associated sRNAs are similar in that they all bind to AtAGO4, suggesting that AtAGO3 participates in plant DNA methylation [30]. AtAGO7 plays an essential role in accelerating the phase change from juvenile to adult stage, and also takes part in the TAS3-based tasiRNA biogenesis [31]. In addition, like AtAGO1, AtAGO7 participates in pathogen defense [17].

AtAGO4 and AtAGO6 bind to different RNA polymerases to carry out RNA-induced DNA methylation orderly [32]. Meanwhile, AtAGO6 also plays a role in transcriptional gene silencing which mediated by RNA in shoot and root meristems [33]. Moreover, AtAGO4 is involved in virus resistance [34, 35]. AtAGO8 is considered as a pseudogene [36] and has no homologous gene in rice and maize. Recently, it was found that AGO8 mediated the induction of primary defense against herbivory in *Nicotiana attenuata* [37]. Nevertheless, AtAGO9 regulates germ cell fate by a non-cell autonomous sRNA pathway [38, 39].

Viral-inducible OsAGO18 sequesters miR168 to ease the repression of rice OsAGO1 by miR168 to enable antiviral defense in the infected rice [40]. OsAGO18 also competes with OsAGO1 for binding to miR528, resulting in the release of the negative regulation of AO gene by OsAGO1-miR528, and then boosts the ROS accumulation to trigger the pathogen defense pathway [41]. However, high level of specific expression of ZmAGO18 in the tapetum and germ cells of maize meiotic anthers [10] is implicated that ZmAGO18 is a negative regulator to determine the inflorescence and axillary meristems by interacting with the regulatory pathway of miR166-HD-ZIP III TF [13]. The expression pattern of ZmAGO1a under five different abiotic stresses indicated that *ZmAGO1a* might play an important role in both development and responses to environmental change as a member of the AGO gene family in maize [42]. Therefore, AGO genes function in the development and pathogen defense of plants, and AGO genes would be up- or down-regulated under virous abiotic stress in rice [11, 43] and cucumber [1].

In this study, we analyzed the differential expressions of 17 ZmAGOs in maize under various abiotic stress treatments, and explored potential drought resistance function of ZmAGO18b, which provided a basis for further understanding and study the stress tolerance mechanism of this important crop.

## Results and discussion

### Expression patterns of ZmAGO genes under heat stress

To investigate the heat stress-responsiveness of *ZmAGO* genes in maize, we transferred the whole maize seedlings into an incubator at 40 °C and then sampled at the six different points (0, 0.5, 1, 2, 4, and 12 h). The results showed that all *ZmAGOs* responded to heat stress.

**Fig. 1:**
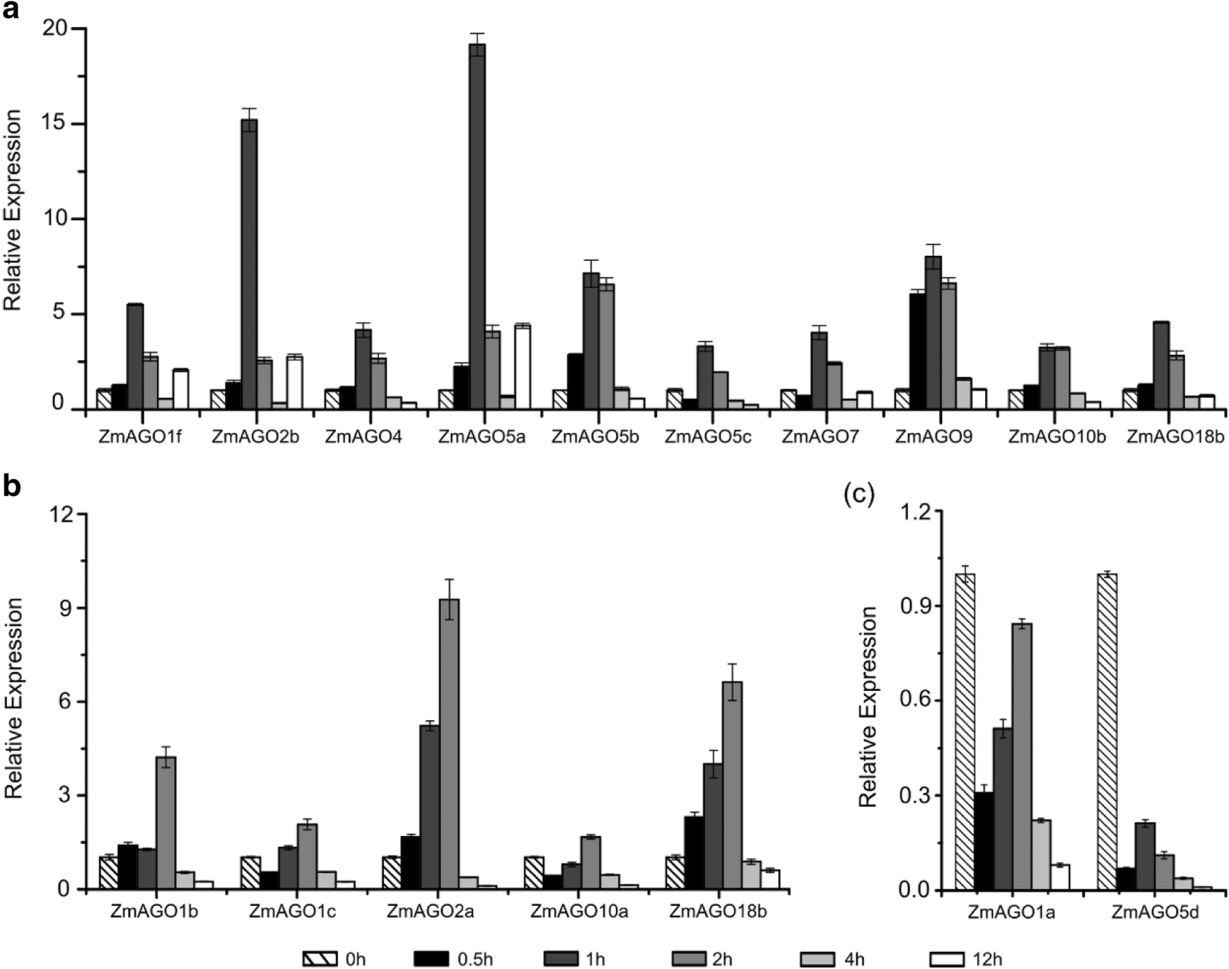
qPCR analysis of ZmAGO genes expression in response to 40 °C treatment at 0, 0.5, 1, 2, 4, 12 h of treatment. ZmActin normalizes the data as an internal control. Y-axis represents relative expression levels

It was found that 10 (*ZmAGO1f*, *ZmAGO2b*, *ZmAGO4*, *ZmAGO5a/b/c*, *ZmAGO7*, *ZmAGO9*, *ZmAGO10b* and *ZmAGO18a*) of the 17 genes were upregulated by this treatment at 1 h and then declined at 2 h, 4 h and 12 h (Fig. 1a). Among these 10 genes, *ZmAGO2b* and *ZmAGO5a* were relatively upregulated strongly at 1 h after treatment. Interestingly, *ZmAGO1b/c*, *ZmAGO2a*, *ZmAGO10a* and *ZmAGO18b* were upregulated and then downregulated at 2 h after heat treatment (Fig. 1b), indicating that each AGO gene has its own function which regulates gene expression in different time periods during abiotic stress. In addition, *ZmAGO1a* and *ZmAGO5d* were downregulated relative to control treatment (Fig. 1c).

### Expression profiling of ZmAGOs in response to cold stress

**Fig. 2:**
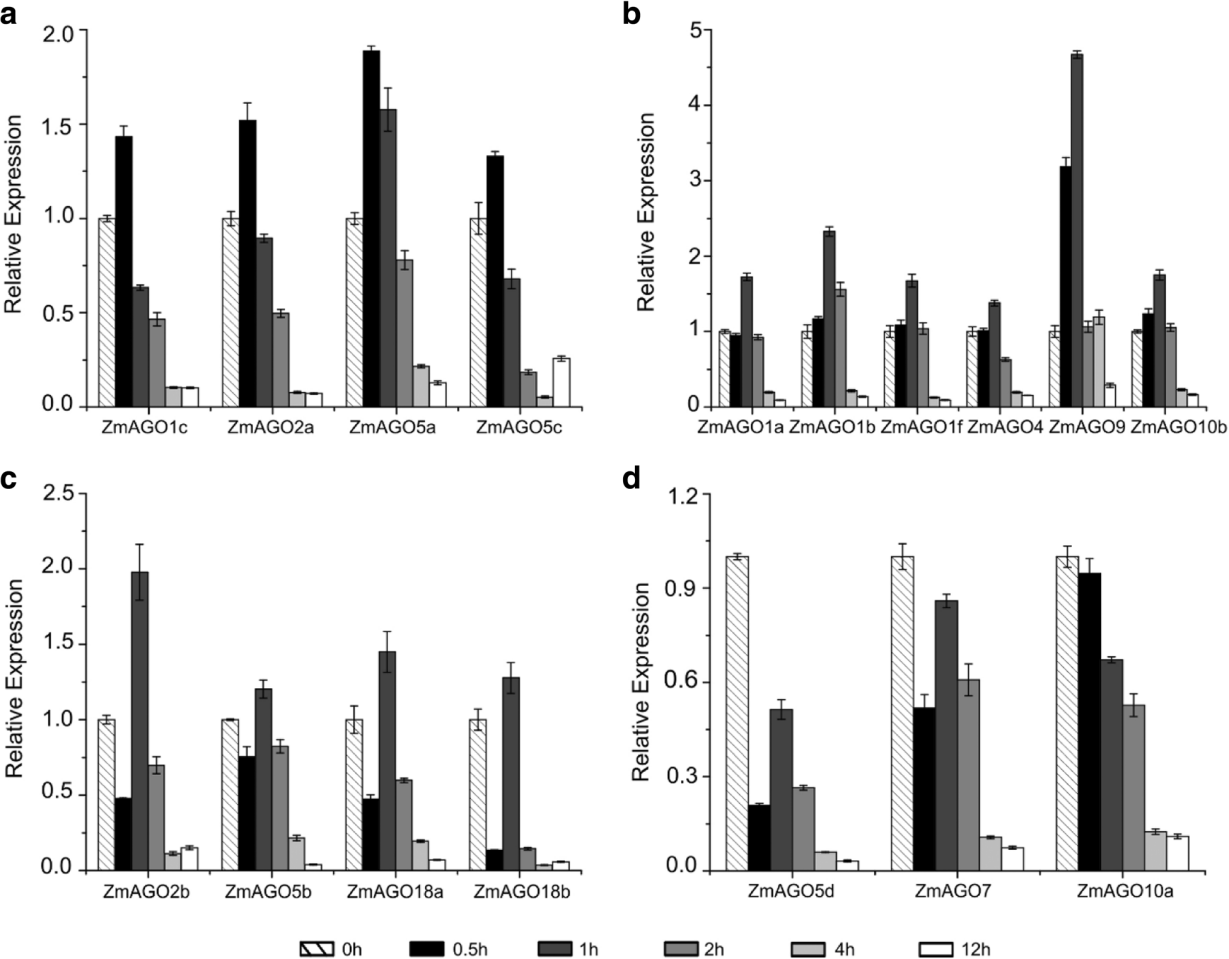
Relative gene expression analysis of ZmAGO genes by qPCR in response to 4 °C treatment at 0, 0.5, 1, 2, 4, 12 h of treatment. ZmActin normalizes the data as an internal control. Y-axis represents relative expression levels

For cold stress, we examined the expression levels of ZmAGO genes in whole seedlings at the six different points (0, 0.5, 1, 2, 4, and 12 h) under 4 °C treatment using qPCR. The results of the relative expression levels of the cold-treated samples are shown in Fig. 2.

Compared to the control, *ZmAGO5d*, *ZmAGO7* and *ZmAGO10a* were downregulated (Fig. 2d), other *ZmAGOs* were upregulated in varying degrees by this treatment. The expression of *ZmAGO1c*, *ZmAGO2a*, *ZmAGO5a* and *ZmAGO5c* were upregulated up to 0.5 h after the cold treatment and then declined gradually at 1, 2, 4 and 12 h (Fig. 2a). *ZmAGO1a*, *ZmAGO1b*, *ZmAGO1f*, *ZmAGO4*, *ZmAGO9* and *ZmAGO10b* increased slightly up to 1 h after treatment and downregulated thereafter (Fig. 2b). *ZmAGO2b*, *ZmAGO5b*, *ZmAGO18a* and *ZmAGO18b* were downregulated at 0.5 h after treatment but then were upregulated at 1 h, followed by a decrease at 2 h, 4 h and 12 h (Fig. 2c). The results showed that almost all *ZmAGOs* were responsive to low temperature. Furthermore, the expression level of all *ZmAGOs* was very low at 4 h after treatment, indicating that maize could resist low temperature for 4 h through various gene expressional regulation.

### Expression analysis of ZmAGO genes by qPCR under NaCl treatment

To discover the responsiveness of *ZmAGO* genes to NaCl treatment, the seedling roots were submerged in a solution of 0.2 M NaCl, and the whole seedlings were sampled at 0, 0.5,1, 2, 4, and 12 h after treatment.

**Fig. 3:**
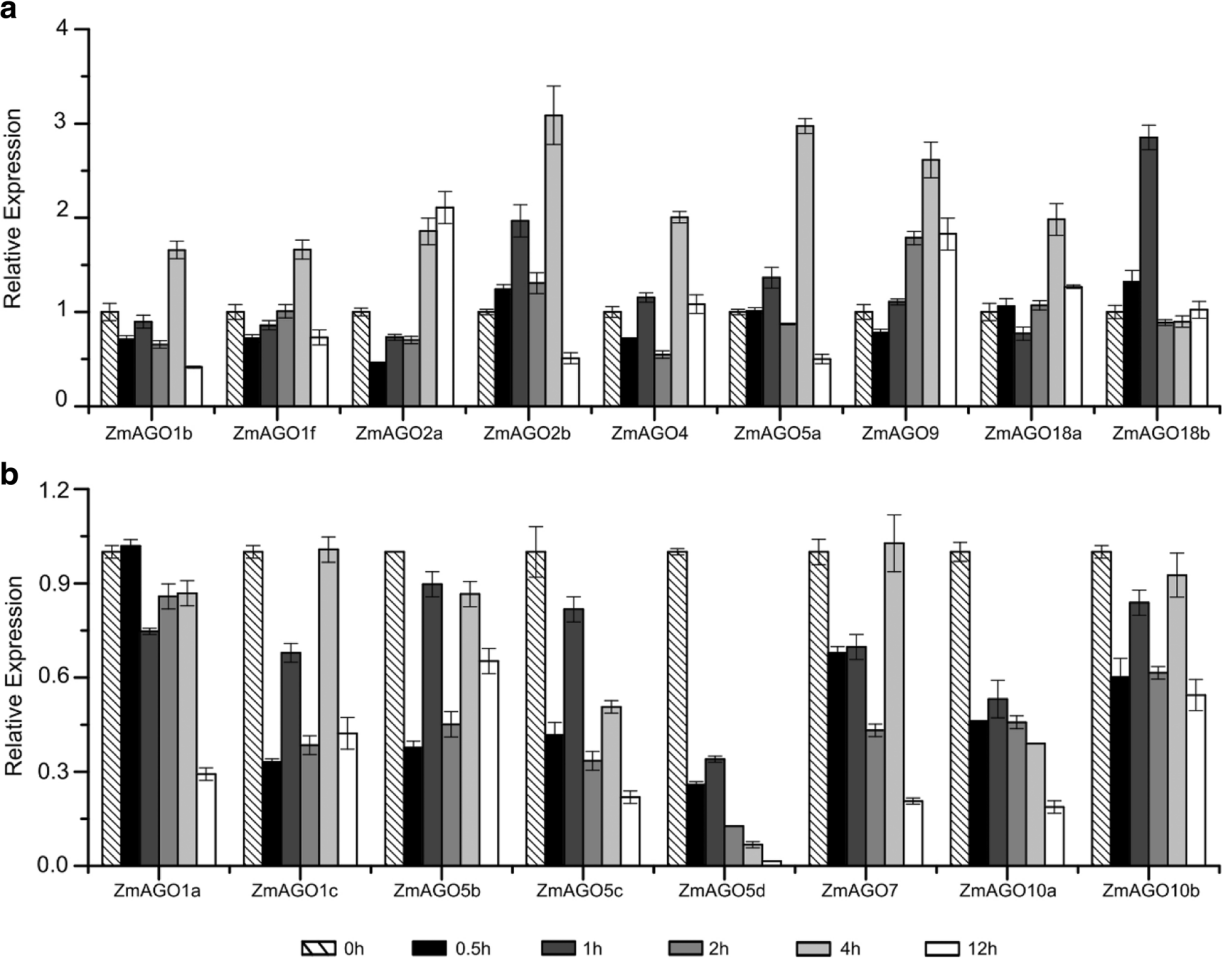
qPCR expression analysis of ZmAGO genes in response to NaCl treatment at 0, 0.5, 1, 2, 4, 12 h of treatment. ZmActin normalizes the data as an internal control. Y-axis represents relative expression levels

Eight genes (*ZmAGO1a/c*, *ZmAGO5b/c/d*, *ZmAGO7* and *ZmAGO10a/b*) out of 17 were downregulated slightly by NaCl treatment (Fig. 3b). *ZmAGO1b/f*, *ZmAGO2b*, *ZmAGO4*, *ZmAGO5a*, *ZmAGO9* and *ZmAGO18a* genes were slightly upregulated until 4 h after NaCl treatment and were downregulated at 12 h after NaCl treatment. But *ZmAGO2a* was upregulated slightly up to 4 and 12 h after NaCl treatment. On the contrary, *ZmAGO18b* was upregulated at 0.5 and 1 h after NaCl treatment and then declined at 2, 4, and 12 h (Fig. 3a). Altogether, the results showed that all ZmAGOs were slightly responsive to NaCl stress.

### Responses of ZmAGOs to drought stress

For drought stress, we pulled the seedling roots out of the soil without water supply, and the whole seedlings were sampled at 0, 0.5,1, 2, 4, and 12 h after drought treatment. The expression analysis showed that all ZmAGO genes were induced to response to the drought treatment.

**Fig. 4:**
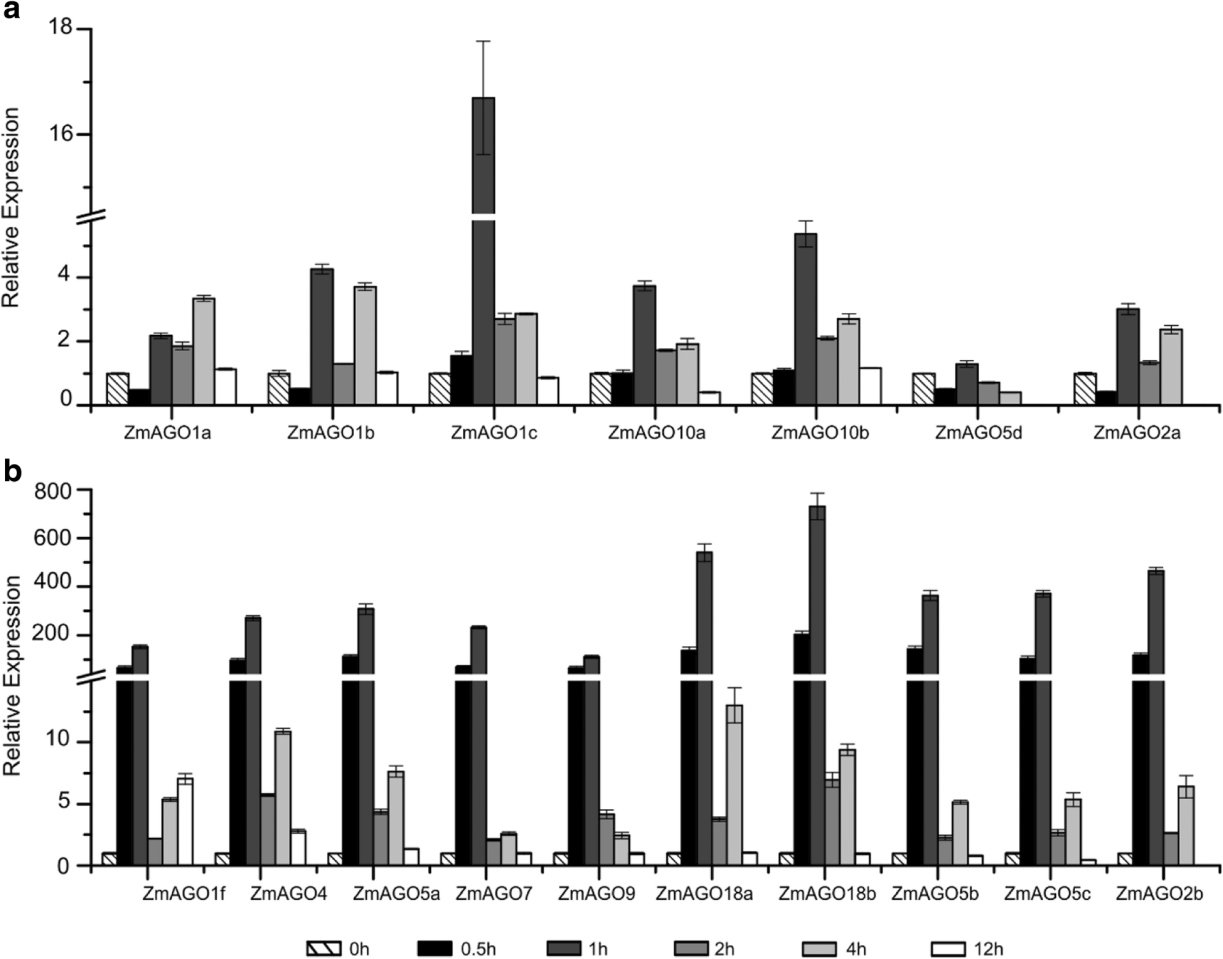
Expression analysis of ZmAGO genes by qPCR in response to drought treatment at 0, 0.5, 1, 2, 4, 12 h of treatment. ZmActin normalizes the data as an internal control. Y-axis represents relative expression levels

According to the response to drought stress, ZmAGO genes can be divided into two categories which are slightly and strongly induced group. *ZmAGO1a/b/c*, *ZmAGO10a/b*, *ZmAGO5d* and *ZmAGO2a* were upregulated slightly at 1 h after the drought stress and then decreased at 2, 4 and 12 h (Fig. 4a). Ten (*ZmAGO1f*, *ZmAGO4*, *ZmAGO5a/b/c*, *ZmAGO7*, *ZmAGO9*, *ZmAGO18a/b* and *ZmAGO2b*) of the 17 genes were significantly upregulated by drought treatment at 1 h and then declined at 2, 4 and 12 h (Fig. 4b). It should be emphasized that ZmAGO18a (539.9-fold upregulated compared to the control) and ZmAGO18b (730.8-fold upregulated compared to the control) were highly induced at 1 h under the drought stress, indicating that the ZmAGO18a/b plays important roles in gene regulation during the drought stress.

### The expression pattern of ZmAGO genes under ABA treatment

To discover the responsiveness of in maize to ABA treatment, we investigated the transcript levels of *ZmAGO* genes in maize whole seedlings under ABA treatment by qPCR. Interestingly, we found that ZmAGO genes responded to the ABA treatment in a time-dependent manner.

**Fig. 5:**
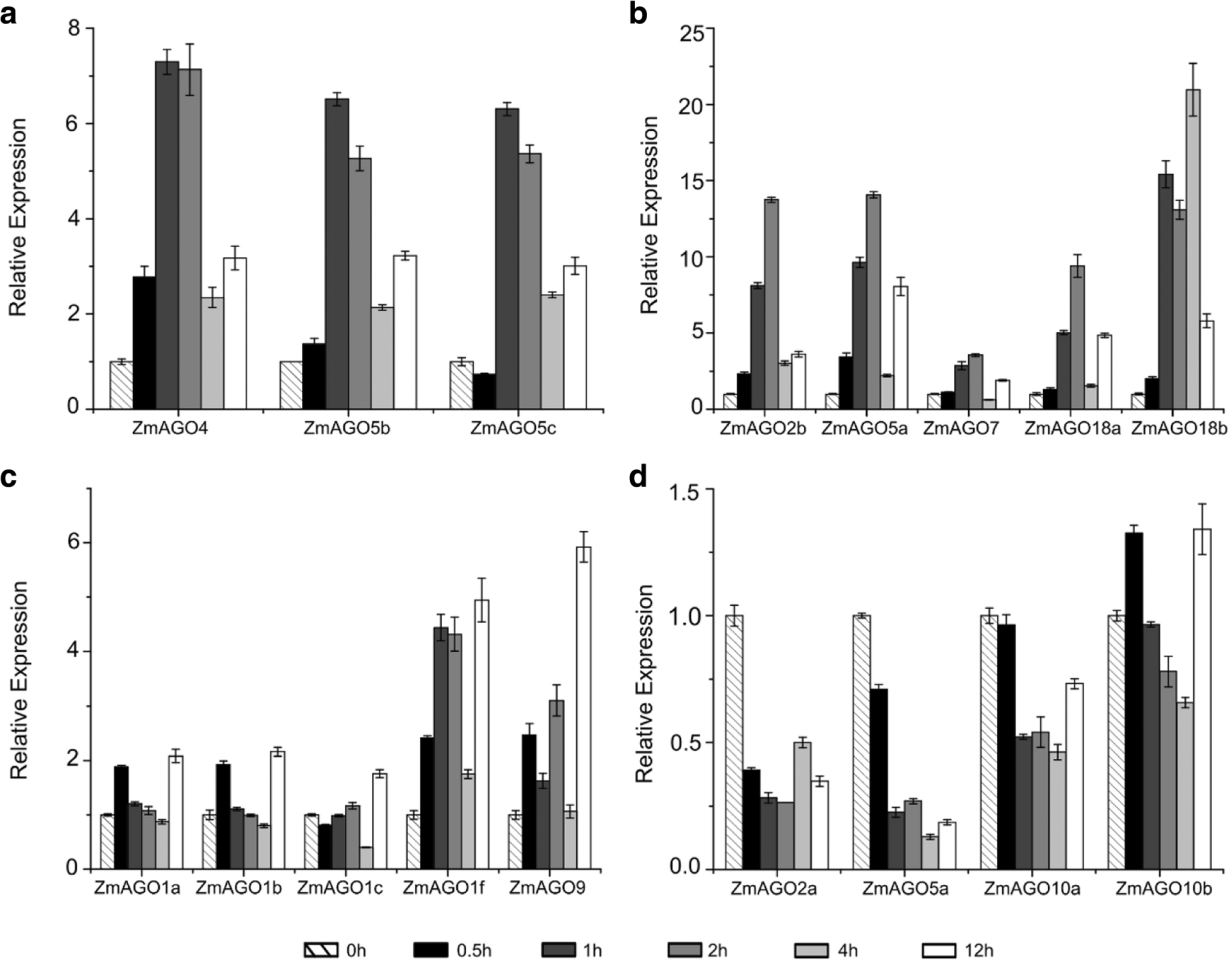
qPCR analysis of ZmAGO genes expression in response to hormone (ABA) treatment at 0, 0.5, 1, 2, 4, 12 h of treatment. ZmActin normalizes the data as an internal control. Y-axis represents relative expression levels

*ZmAGO4* and *ZmAGO5b/c* were upregulated slightly up to 1 h after the ABA treatment and then decreased at 2, 4 and 12 h (Fig. 5a). *ZmAGO2b*, *ZmAGO5a*, *ZmAGO7*, *ZmAGO10b* and *ZmAGO18a* were upregulated gradually at 0.5, 1 and 2 h and then declined at 4 and 12 h. Meanwhile, the highest expression level of *ZmAGO18b* was at 4 h after the ABA treatment and then declined at 12 h (Fig. 5b/d). In the end, *ZmAGO1a/b/c/f* and *ZmAGO9* were maximized induced expression at 12 h (Fig. 5c). But beyond that, *ZmAGO2a*, *ZmAGO5d* and *ZmAGO10a* were slightly downregulated by the ABA treatment compared to the control (Fig. 5d).

In eukaryotic sRNA-based gene silencing pathways, AGO proteins are effectors which function in gene expression regulation and chromatin modification. The majority of studies about plant AGOs revealed their function in plant development and pathogen defense. Some studies reported that plant AGO genes also respond to abiotic stresses. In *Oryza sativa*, nine of 19 OsAGOs (*OsAGO1a/b/c/d*, *OsAGO2*, *OsAGO4a/b*, *OsPNH1* and *OsAGO16*) were upregulated in response to salt, cold and dehydration stresses by microarray-based expression analysis. The samples were collected the 7 days-seedlings which were treated 3 h by the above three stresses [11]. But in the current study, all 17 ZmAGOs were downregulated at 4 h after the cold treatment compared to the control. *ZmAGO1b/f*, *ZmAGO2b*, *ZmAGO4*, *ZmAGO5a*, *ZmAGO9* and *ZmAGO18a* genes were slightly upregulated until 4 h after salt treatment. Another study about OsAGOs expression analysis indicated that six OsAGO genes (*OsAGO1a/d*, *OsAGO3*, *OsAGO7*, *OsAGO13* and *OsAGO16*) were up or down-regulated in response to one or more of the phytohormone 1-Naphthaleneacetic acid (NAA), Kinetin (KT) and Gibberellin A3 (GA3) in seedlings at trefoil stage [43]. Furthermore, these six OsAGOs were sensitive to different hormones and differences existed among varieties [43]. In the present study, 14 out of 17 ZmAGOs were sensitive to ABA and these genes responded to the ABA treatment temporally. Interestingly, the recent research illustrated that AtAGO1 is responsive to hormones and cold stress and triggered by these stimuli to bind to stimuli-responsive genes [19]. In addition to the classical RNAi mechanism, it is suggested that plant AGO genes might facilitate the induction of genes in virous stimuli signaling pathways and the activation of the stimuli responses.

Notably, monocots-specific AGO18 gene is distinctive. OsAGO18 and ZmAGO18 exhibited high level expression during reproductive stage. Whereas OsAGO18 participates the process of pathogen defense [41], ZmAGO18b specifically expresses in meiotic anthers [10] and plays an crucial role in the determinacy of inflorescence and axillary meristems [13]. In this study, ZmAGO18a and ZmAGO18b were significantly reduced in seedlings by drought treatment. This result demonstrates the importance of ZmAGO18s during the drought stress. Further studies should be carried out to investigate mechanisms of the ZmAGO18s function in drought stress, and to provide new insights into the drought resistance.

### Stress responsive elements in the promotor of ZmAGO genes and the phenotype of ZmAGO18b mutant after drought treatment

**Fig. 6:**
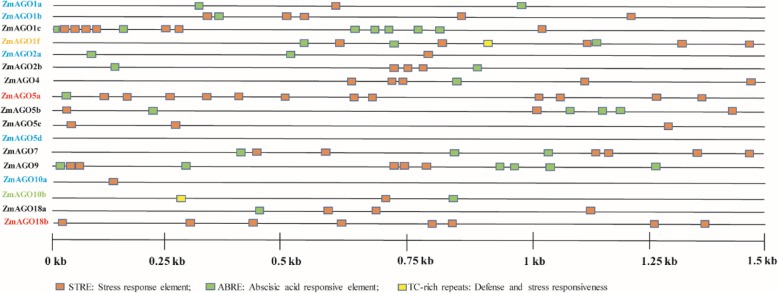
Typical stress and ABA-responsive elements location of ZmAGO gene family

Several *cis*-acting elements related to plant development and response were identified, implicating the possible involvement of AGO gene family in development and stress tolerance (Additional file 1: Table S1). We selected two typical stress responsive elements (STRE and TC-rich repeats) and one ABA responsive element (ABRE) to demonstrate the distribution of *cis*-acting elements in the promotor region (Fig. 6).

**Fig. 7:**
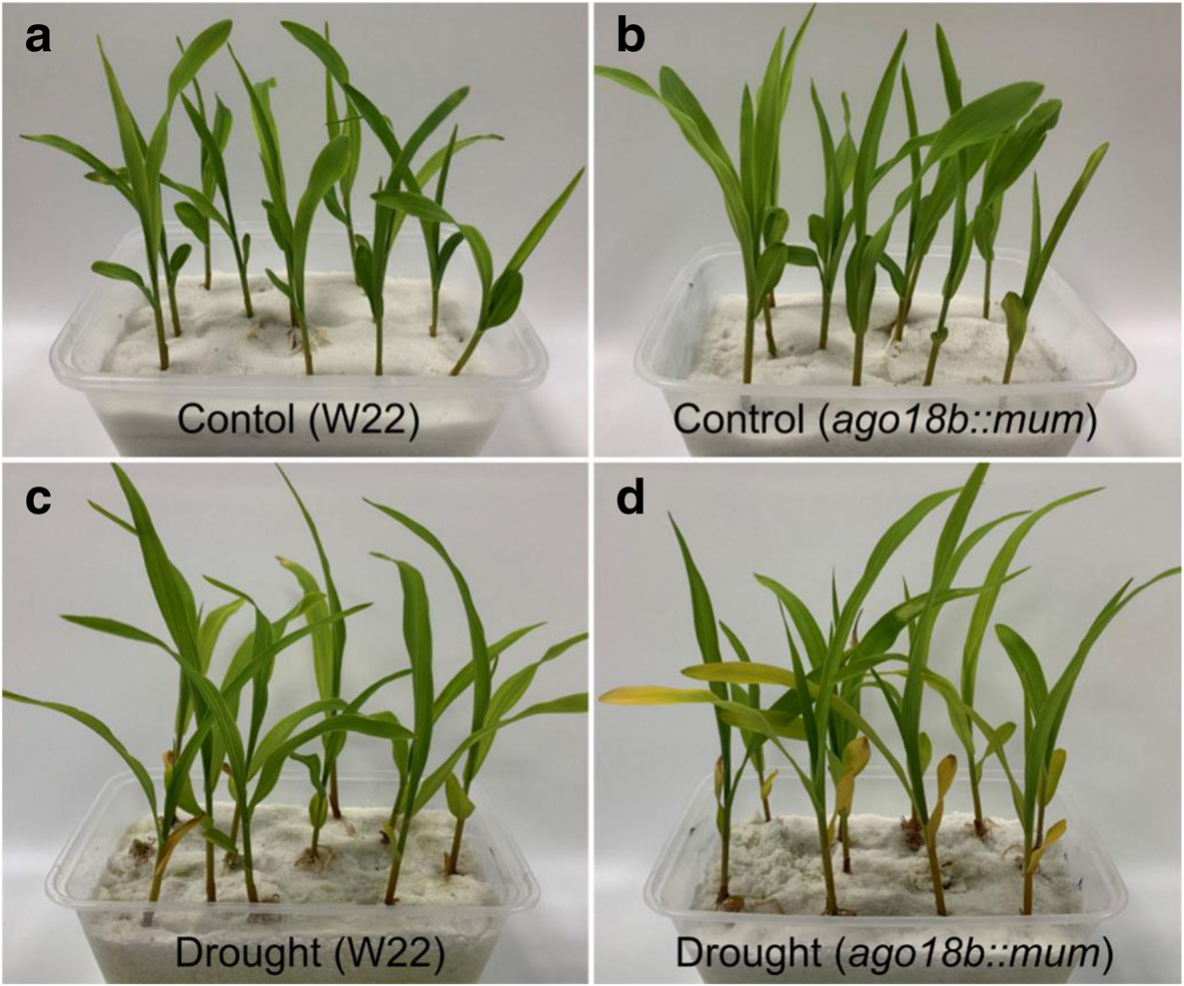
The phenotype of W22 and *ago18b:mum* after 24 h of drought treatment and corresponding control. **a** W22 without drought treatment. **b** *ago18b:mum* without drought treatment. **c** W22 with drought treatment. **d** *ago18b:mum* with drought treatment

As shown in Fig. 6, STRE element is abundant in promotor region of the majority of AGO gene family. In addition, we found that genes with the same expression pattern are likely to have similar elements. For example, *ZmAGO18b* and *ZmAGO5a* were specifically expressed in meiotic tassel [10] and they were all responsive to drought and heat in this study, and the largest number of STRE elements were distributed in their promotor region, 8 and 12, respectively. These data suggested that these genes might have potential function of resistance to stress during the meiosis of tassel. *ZmAGO1f* and *ZmAGO10b* are highly expressed in immature tassel, and they have unique TC-rich repeats element. The ABA-insensitive genes (*ZmAGO1a/b*, *ZmAGO2a*, *ZmAGO5d* and *ZmAGO10a/b*) have fewer ABRE, but not all. To further determine the drought response of ZmAGO18b gene, we observed the phenotype of *Mutate*-mediated mutant of ZmAGO18b (*ago18b:mum*) described previously [13]. The results showed that the mutant leaves displayed more severe yellowing after 24 h of drought treatment (Fig. 7), indicating the drought resistance function of ZmAGO18b gene. We will further study the drought resistance mechanism of ZmAGO18b gene.

## Conclusions

In summary, this work is the first report on expression analysis of all ZmAGO genes in maize under heat, cold, salt, drought and ABA stress treatments. We discovered that various *ZmAGO* genes respond to different abiotic stress treatments. And ZmAGO18b may have potential drought resistance function, which prompts us to further study its drought resistance mechanism. According to results of the present study, it should consider as a basis for intensive functional research of ZmAGO genes during abiotic stress.

## Materials and methods

### Plant materials and treatments

Seeds of maize (*Zea mays* L. cvB73) were grown in potting soil under greenhouse conditions at 25 °C with an 8 h dark and 16 h light (Department of Molecular Biology of Hubei University of Arts and Science, Xiangyang, China). Two-week-old seedlings growing synchronously were selected to impose different abiotic stress treatments, which including heat, cold, salinity, drought and abscisic acid (ABA). Gene expression was analyzed at different time points of treatments (0, 0.5, 1, 2, 4, and 12 h). We select three individual and whole seedlings as three independent biological replicates at each treatment point. For cold and heat treatments, potted maize seedlings were respectively incubated at 4 °C and 40 °C. Salinity treatment was applied by submerging seedling roots in a 0.2 M NaCl solution. To impose the drought treatment, we gently pulled the whole maize seedlings out of the soil. In order to remove the adhering soil, we washed their roots carefully with fresh water. Then the seedlings were incubated on a dry paper towel at 25 °C, water was not supplied throughout the treatment process. For ABA treatment, seedlings roots were submerged in a solution of 0.1 mM ABA. Rapid freezing of samples with liquid nitrogen and preservation at − 80 °C for RNA isolation.

Seeds of W22 and Mutate-mediated mutant of ZmAGO18b (ago18b:mum) which described in the previous study [13] were also grown under the conditions described above for drought treatment. The phenotype of these materials after drought treatment for 24 h were observed.

### RNA extraction and qPCR

Total RNA from the different stress treated samples was extracted using Trizol (Invitrogen, Carlsbad, CA, USA). Genomic DNA contaminants were removed from the RNA by treating the RNA with DNaseI (TaKaRa Biotech, Dalian, China). A Qubit 2.0 (Invitrogen) was used to measure the RNA concentration in each sample. An oligo (dT) primer and M-MLV (Invitrogen) reverse transcriptase was used to synthesize first-strand cDNAs from RNA following the manufacture’s protocol.

Gene-specific primers reference to previous described for the qPCR expression analysis [10]. ZmActin (NM_001155179) was used as the internal control and was amplified with the primers 5′- TACGAGATGCCTGATGGTCAGGTCA − 3′ and 5′- TGGAGTTGTACGTGGCCTCATGGAC -3′. QPCR was carried out on the Bio-RAD CFX96 using the SYBR FAST qPCR Kit Master Mix (2x) LightCycler (KAPA, USA). The qPCR was conducted in 20 μL reaction volumes consisting of 3.0 μL diluted cDNA, 1.0 μL forward and reverse primers (10 μM), 10 μL SYBR FAST qPCR Kit Master Mix (2x) LightCycler (KAPA, USA) and 5 μL double-distilled water. The qPCR conditions were as follows: pre-denaturation for 300 s at 95 °C, 40 cycles at 95 °C for 10 s, 58 °C or 60 °C for 20 s. Three biological replicates were performed. The relative gene expression was calculated using the 2^−∆∆Ct^ method [44] in the EXCEL software.

### Cis-regulatory elements analysis of ZmAGOs promotor

To analyze the stress responsive elements of ZmAGO genes, we identified putative cis-regulatory elements of about 5 to 10 bp in around 1500-bp upstream from the start codon (ATG) of 17 ZmAGO genes using the PlantCARE web tool (http://bioinformatics.psb.ugent.be/webtools/plantcare/html/).

## Additional file

Additional file 1: **Table S1.** Putative *cis*-elements of more than 5 bp identified in 17 ZmAGO genes using PlantCARE database. (XLSX 64 kb)

## Data Availability

The datasets used and/or analysis in the current study can be obtained from the corresponding author according to reasonable requirements.

## Abbreviations

ABA: Abscisic acid
AGO: Argonaute
qPCR: Quantitative PCR
RISC: RNA-induced silencing complex
ROS: Reactive oxygen species
sRNA: Small RNA
tasiRNA: Trans-acting small interfering RNA
